# Individualized Autoregulation-Derived Cerebral Perfusion Targets in Aneurysmal Subarachnoid Hemorrhage: A New Therapeutic Avenue?

**DOI:** 10.1177/08850666241252415

**Published:** 2024-05-05

**Authors:** Teodor Mikael Svedung Wettervik, Anders Hånell, Timothy Howells, Elisabeth Ronne-Engström, Anders Lewén, Per Enblad

**Affiliations:** 1 Department of Medical Sciences, Section of Neurosurgery, 8097 Uppsala University, Uppsala, Sweden

**Keywords:** cerebral perfusion pressure, neurointensive care, optimal cerebral perfusion pressure, outcome, pressure reactivity index, subarachnoid hemorrhage

## Abstract

**Background:** Cerebral perfusion pressure (CPP) is an important target in aneurysmal subarachnoid hemorrhage (aSAH), but it does not take into account autoregulatory disturbances. The pressure reactivity index (PRx) and the CPP with the optimal PRx (CPPopt) are new variables that may capture these pathomechanisms. In this study, we investigated the effect on the outcome of certain combinations of CPP or ΔCPPopt (actual CPP-CPPopt) with the concurrent autoregulatory status (PRx) after aSAH. **Methods:** This observational study included 432 aSAH patients, treated in the neurointensive care unit, at Uppsala University Hospital, Sweden. Functional outcome (GOS-E) was assessed 1-year postictus. Heatmaps of the percentage of good monitoring time (%GMT) of PRx/CPP and PRx/ΔCPPopt combinations in relation to GOS-E were created to visualize the association between these variables and outcome. **Results:** In the heatmap of the %GMT of PRx/CPP, the combination of lower CPP with higher PRx values was more strongly associated with lower GOS-E. The tolerance for lower CPP values increased with lower PRx values until a threshold of −0.50. However, for decreasing PRx below −0.50, there was a gradual reduction in the tolerance for lower CPP. In the heatmap of the %GMT of PRx/ΔCPPopt, the combination of negative ΔCPPopt with higher PRx values was strongly associated with lower GOS-E. In particular, negative ΔCPPopt together with PRx above +0.50 correlated with worse outcomes. In addition, there was a transition toward an unfavorable outcome when PRx went below −0.50, particularly if ΔCPPopt was negative. **Conclusions:** The PRx levels influenced the association between CPP/ΔCPPopt and outcome. Thus, this variable could be used to individualize a safe CPP-/ΔCPPopt-range.

## Introduction

Patients with aneurysmal subarachnoid hemorrhage (aSAH) commonly develop cerebral vasospasm and exhibit chronic arterial hypertension, which make them vulnerable to developing cerebral ischemia despite “normal” cerebral perfusion pressure (CPP) levels.^1,2^ However, what constitutes a sufficient CPP may differ between aSAH patients and change over time, due to the dynamic course of cerebral pressure autoregulation and vasospasm.^3^ Hence, there is an interest in finding ways to monitor the cerebral pressure autoregulatory status as a means to individualize CPP management in aSAH.^2^

**Figure 1.**
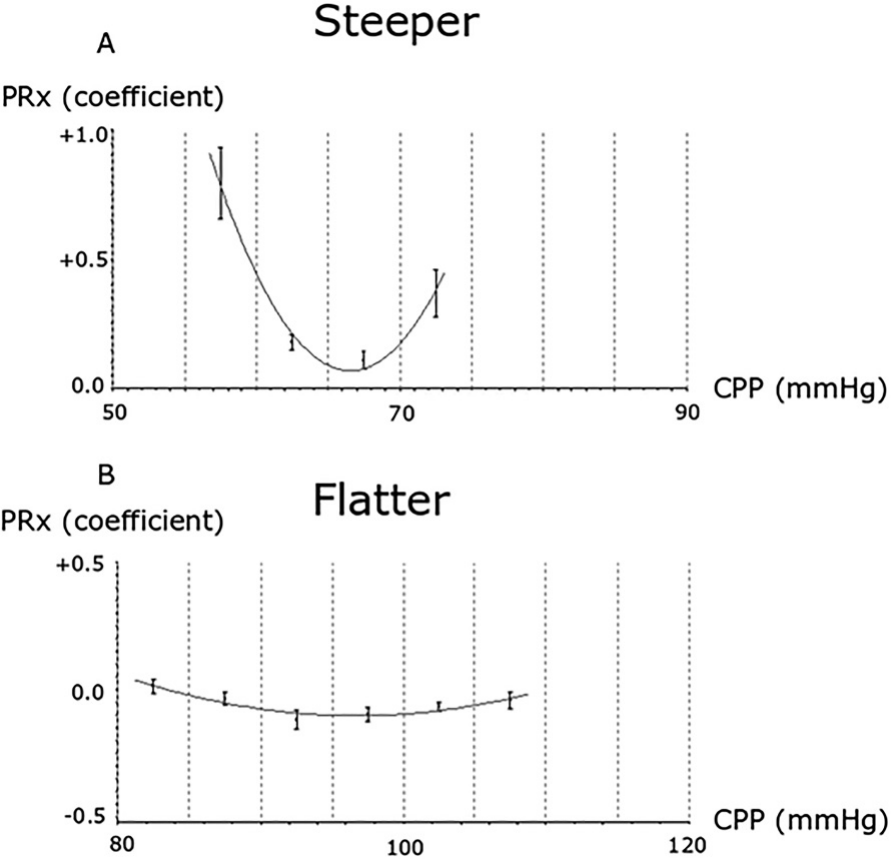
(A-B) CPPopt curve shape variation—2 examples. The figure illustrates 2 different CPPopt curves. (A) A scenario with a steep U-shape, possibly indicating a stronger indication to keep CPP close to CPPopt to maintain a balanced CBF. (B) A flat curve with generally low values, where the cerebral pressure autoregulation appears intact and CBF is likely maintained over the entire CPP range.

Abbreviations: CBF, cerebral blood flow; CPP, cerebral perfusion pressure; CPPopt, optimal CPP.

The pressure reactivity index (PRx) is a method for continuous cerebral pressure autoregulation monitoring and is defined as the 5-minute correlation of 10 s-values of arterial blood pressure (ABP) and intracranial pressure (ICP),^4^ where negative values indicate preserved cerebral pressure autoregulation and vice versa. PRx often varies with CPP in a U-shaped fashion and the CPP at the lowest point of this curve has been denoted as optimal (CPPopt).^5,6^ CPPopt is hypothesized to be a better CBF surrogate than absolute CPP levels in traumatic brain injury (TBI),^5–11^ but it remains elusive if this concept is valid in aSAH. Some aSAH studies have found that CPP below CPPopt is associated with lower CBF^12^ and brain hypoxia^13^ whereas others report a lack of association between ΔCPPopt (actual CPP-CPPopt) and functional outcomes.^3,14^ An important issue with the CPPopt concept is if targeting ΔCPPopt within a narrow mm Hg interval is too crude, considering that the curve shape (flat/sharp) and the absolute PRx values (high/low) differ between patients and over time (Figure 1).

To better understand how the cerebral pressure autoregulatory status may influence the effect of CPP on the brain after aSAH, we aimed to study combined insults of PRx together with CPP and ΔCPPopt versus outcome. We hypothesized that high PRx combined with low CPP or negative ΔCPPopt would be particularly correlated with worse outcomes since the loss of pressure autoregulation with low perfusion pressure strongly indicates detrimental cerebral ischemia. We also hypothesized that the CPPopt curve shape and its corresponding absolute PRx values would be important so that exceeding a certain reactivity-defined threshold (eg, a negative ΔCPPopt with PRx > 0.2) would better correlate with the functional outcome than deviations from ΔCPPopt based on mm Hg-thresholds.

## Materials and Methods

### Study Design and Patients

This was an observational single-center study. aSAH patients who were above 15 years old and treated at the neurointensive care (NIC) unit, Uppsala University Hospital, Sweden, between 2008 and 2018, were eligible for inclusion. Of 956 aSAH patients, 524 were excluded (420 without ICP monitoring, 97 with < 24 h of CPPopt data during the first 10 days, and 7 without available outcome data), leading to a final study population of 432 patients.

### Management Protocol

The management protocol has been described in detail in a previous study^15^ and remained unchanged throughout the study period. Treatment goals were ICP ≤ 20 mm Hg, CPP ≥ 60 mm Hg, P_a_O_2 _> 12 kPa, arterial glucose 5 to 10 mmol/L (mM), electrolytes within normal ranges, and body temperature < 38 °C. Nimodipine was given to all patients after admission. Ruptured aneurysms were treated with early occlusion using clip ligation or endovascular intervention. All unconscious patients (Glasgow Coma Scale Motor score < 6) were intubated, mechanically ventilated, sedated, and received ICP monitoring. The ICP monitor of choice was external ventricular drainage (EVD), otherwise, an intraparenchymal monitor was used if the ventricles were small. The EVD was typically opened at 15 mm Hg if the ICP was high. Thiopental infusion and decompressive craniectomy were last-tier ICP treatments. Arterial hypotension was primarily treated with clear fluids and colloids (albumin 20%), while dobutamine/norepinephrine was used as a second choice to maintain CPP targets. Delayed ischemic neurological deficits (DINDs) were defined as a delayed neurological deterioration in consciousness or onset of focal neurological deficits, that could not be explained by other causes, such as rebleeding, acute hydrocephalus, or meningitis. If DIND was diagnosed, HHH (hypertension, hypervolemia, and hemodilution) therapy were initiated.^16^

### Functional Outcome

Functional outcome was assessed 1-year postictus, using the Glasgow Outcome Scale-Extended (GOS-E).^17,18^ The scale contains 8 categories and ranges from death (1) to upper good recovery (8). The assessment was done with the patient or their next-of-kin by trained staff using structured telephone interviews. Favorable and unfavorable outcomes were defined as GOS-E 5 to 8 and 1 to 4, respectively.

### Acquisition of Physiological Data

The physiological data were collected at 100 Hz using the Odin software.^19^ ICP was monitored with an external ventricular drainage system (EVD; HanniSet, Xtrans, Smith Medical GmbH, Glasbrunn, Germany) or an intraparenchymal probe (Codman ICP Micro-Sensor, Codman & Shurtleff, Raynham, MA). ABP was measured in the radial artery at the heart level. PRx was calculated in retrospect as the moving 5-minute correlation of 10 s averages of ICP and ABP.^4,11^ CPPopt was also calculated in retrospect as the CPP with the lowest PRx in the last 4 h.^5,6,11^ The median amount of available CPP data was 8 (IQR 6-9) days during the first 10 days postictus. CPPopt could be calculated during 54% of the total monitoring time with available CPP data, similar to previous studies.^3,11^ PRx and CPPopt were not available bedside and were not incorporated in the decision-making. The data were analyzed during the first 10 days after ictus, primarily in the early phase (days 1-3) and the vasospasm phase (days 4-10), separately.

### Analysis of Cerebral Perfusion Variables

CPP and ΔCPPopt were analyzed within certain thresholds (eg, CPP < 60 mm Hg and ΔCPPopt < **− **5 mm Hg) and in combination with PRx (eg, CPP < 60 mm Hg and PRx > 0.2) as the percentage of good monitoring time (%GMT) within and outside these boundaries. GMT was defined as the total monitoring time subtracted by the time when the data acquisition was interrupted (eg, when the patients left the NIC for surgery) and after the removal of artefactual values.

For absolute CPP values, the %GMT was analyzed below 60 mm Hg, between 60 and 80 mm Hg, and above 80 mm Hg. The lower limit at 60 mm Hg was used in accordance with our management protocol, 60 to 80 mm Hg was a grey zone between low and high, and 80 mm Hg and above was considered as high CPP. In addition, the same CPP intervals were analyzed in combination with being above or below certain PRx values (0.00, + 0.20, + 0.40, and + 0.60). PRx below/above 0 reflects the transition from negative to positive ABP/ICP correlation. Previously, a PRx threshold around + 0.20 to + 0.40 has been estimated to reflect the limit of autoregulation.^20,21^ Therefore, we considered it most interesting to explore different PRx thresholds within the interval 0.00 to + 0.60 with a + 0.20 change in PRx for each step.

**Figure 2.**
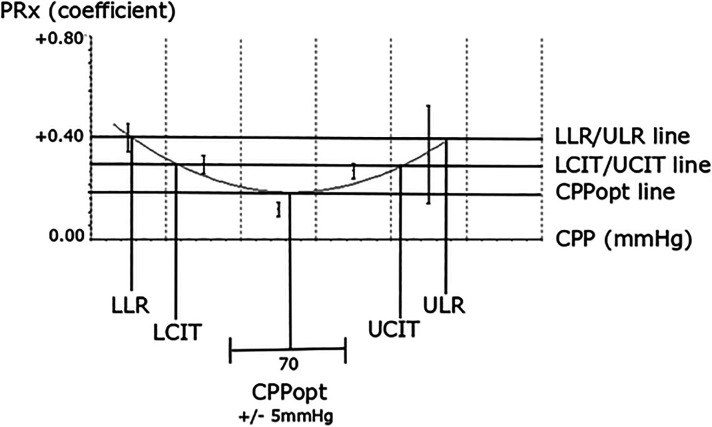
ΔCPPopt in relation to mm Hg, LLR/ULR, and LCIT/UCIT thresholds. The figure illustrates a CPPopt curve and the definition of the LLR for CPP below CPPopt when PRx exceeds + 0.40 and of the ULR for CPP above CPPopt when PRx exceeds + 0.40. It also illustrates LCIT when CPP was below CPPopt and PRx had increased with + 0.10 from CPPopt and UCIT when CPP was above CPPopt and PRx had increased with + 0.10. As outlined, the ΔCPPopt within ± 5 mm Hg, LLR/ULR, and LCIT/UCIT may differ.

Abbreviations: CPP, cerebral perfusion pressure; CPPopt, optimal CPP; ΔCPPopt, CPP-CPPopt; LCIT, lower confidence interval threshold; LLR, lower limit of reactivity; UCIT, upper confidence interval threshold; ULR, upper limit of reactivity.

Furthermore, we studied CPPopt, that is, the CPP with the relatively best PRx, and explored ΔCPPopt based on different threshold definitions. First, the %GMT of ΔCPPopt deviations from certain mm Hg-derived thresholds was calculated (Figure 2). The %GMT of ΔCPPopt < **− **5 mm Hg (hypoperfusion), ΔCPPopt ± 5 mm Hg (optimal), and ΔCPPopt > 5 mm Hg (hyperperfusion) were analyzed, consistent with the COGiTATE trial.^22^ In addition to the ΔCPPopt cut-off at 5 mm Hg, similar analyses were conducted as we explored thresholds at 10, 15, and 20 mm Hg. Second, the %GMT of ΔCPPopt deviations from certain reactivity-derived thresholds were analyzed, that is, below the “the lower limit of reactivity (LLR),” above the “upper limit of reactivity (ULR),” and within these thresholds (Figure 2), similar to previous studies.^20,21,23^ This was done to take into account that negative or positive ΔCPPopt may be dangerous only after a certain autoregulatory threshold is exceeded. We explored the same PRx-thresholds for the LLR/ULR as mentioned above, that is, PRx above 0, + 0.20, + 0.40, and + 0.60 as the reactivity thresholds. For example, the %GMT for ΔCPPopt below the LLR with a PRx threshold at + 0.60 was then defined as the percentage of time with a negative ΔCPPopt when PRx was above + 0.60. Third, the %GMT of ΔCPPopt deviations based on a certain PRx increase from the nadir of the CPPopt curve was calculated, that is, ΔCPPopt below the lower confidence interval threshold (LCIT), above the upper confidence interval threshold (UCIT), and within these thresholds (Figure 2). This was done to both take into account the CPP with the lowest PRx and set the CPP boundaries according to the curve shape based on how large PRx increases that could be tolerated. We started exploring small PRx ranges from + 0.025 and gradually increased the interval to + 0.10, + 0.20, + 0.40, and + 0.60. For example, the %GMT for LCIT with a PRx increase at + 0.10 was then defined as the percentage of time with a negative ΔCPPopt when PRx had increased with + 0.10 or more.

### Visualization of Combined Insults

To study the combined role of absolute PRx/CPP thresholds, 2-dimensional plots were created, which illustrated the correlation between %GMT of various PRx/CPP combinations in relation to GOS-E. This method has been developed by our group and has been described in detail in a previous study.^23^ The %GMT during the first 10 days after ictus for combinations of PRx (range −1 to + 1 with a 0.05 resolution) and CPP values (range 40-120 mm Hg, with a 2 mm Hg resolution), yielding a grid of 1600 cells (40 × 40), was calculated for all patients and analyzed in relation to GOS-E using the Spearman correlation. To reduce high-frequency noise, each pixel was divided into 3 × 3 smaller pixels followed by a Gaussian smoothing (standard deviation = 2). The final values for each pixel were translated into the jet color range (red to blue) with red/blue color indicating a negative/positive association with GOS-E. The jet color scale was limited to ± 0.30 correlation coefficient range due to the moderate correlation strength. Pixels with less than 5 patients with at least 5 min of monitoring time were colored as white. Furthermore, a density plot was created to visualize the frequency of the %GMT for certain combinations of PRx and CPP. The resulting numbers were divided by the highest count within the grid to yield density values ranging from 0 to 1 for each cell in the grid. A Gaussian smoothing was applied also here and the final values were then transformed to colors using the jet color scale and plotted. Similar plots were created for the early phase and the vasospasm phase. Furthermore, similar plots were done with ΔCPPopt, instead of CPP, in combination with PRx in relation to GOS-E.

### Statistical Analyses

Nominal variables were presented as numbers (proportions) and ordinal/continuous variables as medians (interquartile range [IQR]). Differences in ICP, PRx, CPP, and CPPopt in the early phase and the vasospasm phase were analyzed with the Wilcoxon test. The association between the %GMT within/outside the various threshold combinations for the perfusion variables CPP and ΔCPPopt, in both the early phase and the vasospasm phase, were calculated in relation to GOS-E using the Spearman test. Multiple logistic regressions with unfavorable outcomes as the dependent variable and age, WFNS grade, ICP, DINDs, and the %GMT of different perfusion variables were conducted. We selected the former 4 variables to adjust for major prognostic factors of demography (age), primary brain injury (WFNS grade), intracranial hypertension (ICP), and complications (DIND). In separate regressions, we explored different perfusion variable concepts and chose the threshold with the strongest association with the outcome for each concept. Thus, we analyzed %GMT of absolute CPP values without taking into account PRx, %GMT of absolute CPP values in combination with PRx, as well as the ΔCPPopt within/outside a certain mm Hg threshold, within/outside a certain reactivity threshold (LLR/ULR), and within/outside a certain PRx confidence interval (LCIT/UCIT). As this was an exploratory study, we abstained from adjustments for multiple corrections. A *P*-value < .05 was considered statistically significant. The statistical analyses were conducted in RStudio software (version 2022.12.0).^24^

### Ethics

All procedures performed in the studies involving humans were in accordance with the ethical standards of the institutional and national research committee and with the 1964 Helsinki Declaration and its later amendments. The study was approved by the Swedish Ethical Review Authority (2020-05462). Written informed consent was obtained by the closest relatives or from the patients after recovery. In those cases when the relatives or the patients had not responded at follow-up, consent was waived if the relatives or the patient had not objected to taking part in the study (2020-05462).

## Results

### Demography, Admission Variables, Treatments, and Clinical Outcome

In this cohort of 432 aSAH patients, the median age was 59 (IQR 51-67) years and 139/293 (32/68%) were male/female. The median WFNS grade was 4 (IQR 2-4) and the median Fisher grade was 4 (IQR 3-4). The aneurysm was located in the anterior/posterior circulation in 356/76 (82/18%) patients. Most patients were treated with endovascular aneurysm occlusion (n = 303 [70%]), while 118 (27%) patients were operated with clip ligation, 3 (1%) with both treatment modalities, and 8 (2%) cases could not be treated. One hundred (23%) developed DIND. Forty-two (10%) patients received thiopental infusion and 44 (10%) were operated with DC as last-tier treatments for intracranial hypertension. At 1-year follow-up, 85 (20%) were deceased and 138 (32%) had recovered favorably.

### Cerebral Physiology During the Early Phase and the Vasospasm Phase

The median ICP remained unchanged from the early phase to the vasospasm phase (12 [IQR 10-14] vs 12 [IQR 10–14] mm Hg, *P* = .51). The median PRx increased significantly (*P* < .001) from 0.18 (IQR 0.07-0.28) in the early phase to 0.21 (IQR 0.09-0.33) in the vasospasm phase. The median CPP also increased significantly (*P* < .001) from 74 (IQR 70-80) mm Hg in the early phase to 82 (IQR 76-88) mm Hg in the vasospasm phase, similar to CPPopt, which increased (*P* < .001) from 77 (IQR 72-83) mm Hg in the early phase to 81 (IQR 75-87) mm Hg in the vasospasm phase.

### Absolute Cerebral Perfusion Pressure and Pressure Reactivity Index Values in Relation to Functional Outcome

A higher %GMT of CPP < 60 mm Hg in the early phase and the vasospasm phase correlated with lower GOS-E, whereas a higher %GMT of CPP > 80 mm Hg in these phases correlated with higher GOS-E (Table 1 and Supplemental Table 1). The combination of CPP < 60 mm Hg together with PRx > 0.60 held the strongest correlation with lower GOS-E both in the early phase and the vasospasm phase.

**Table 1.** Association Between Absolute PRx and CPP Thresholds in the Early Phase and the Vasospasm Phase in Relation to GOS-E: A Spearman Correlation Analysis.

Variables (%GMT)	Phase	All CPAs	Preserved CPA				Lost CPA			
			PRx < 0.00	PRx < + 0.20	PRx < + 0.40	PRx < + 0.60	PRx > 0.00	PRx > + 0.20	PRx > + 0.40	PRx > + 0.60
CPP < 60 mm Hg	Early phase	***−0***. ***14***^b^	−0.10	−0.09	−0.10	***−0***. ***11***^a^	***−0***. ***16***^b^	***−0***. ***16***^b^	***−0***. ***18***^c^	***−0***. ***20***^c^
	Vasospasm phase	***−0***. ***27***^c^	***−0***. ***18***^c^	***−0***. ***20***^c^	***−0***. ***21***^c^	***−0***. ***22***^c^	***−0***. ***29***^c^	***−0***. ***30***^c^	***−0***. ***32***^c^	***−0***. ***33***^c^
CPP 60-80 mm Hg	Early phase	−0.06	−0.05	−0.04	−0.03	−0.03	0.06	−0.06	−0.06	−0.08
	Vasospasm phase	***−0***. ***16***^b^	−0.07	−0.07	−0.07	***−0***. ***09***	***−0***. ***15***^b^	***−0***. ***17***^c^	***−0***. ***19***^c^	***−0***. ***22***^c^
CPP > 80 mm Hg	Early phase	***0***. ***12***^a^	0.08	0.10	***0***. ***11***^a^	***0***. ***12***^a^	***0***. ***13***^a^	***0***. ***13***^a^	***0***. ***11***^a^	0.08
	Vasospasm phase	***0***. ***23***^c^	***0***. ***16***^c^	***0***. ***21***^c^	***0***. ***24***^c^	***0***. ***25***^c^	***0***. ***22***^c^	***0***. ***19***^c^	***0***. ***13***^b^	0.06

Abbreviations: CPA, cerebral pressure autoregulation; CPP, cerebral perfusion pressure; GMT, good monitoring time; GOS-E, Glasgow Outcome Scale-Extended; PRx, pressure reactivity index.

Preserved and lost CPA were explored according to different operational definitions as below and above 0.00, + 0.20, + 0.40, and + 0.60, respectively, where higher values indicate a more disturbed CPA.

^a^ *P* < .05, ^b^ *P* < .01, ^c^ *P* < .001. Bold and italics indicate statistical significance.

**Figure 3.**
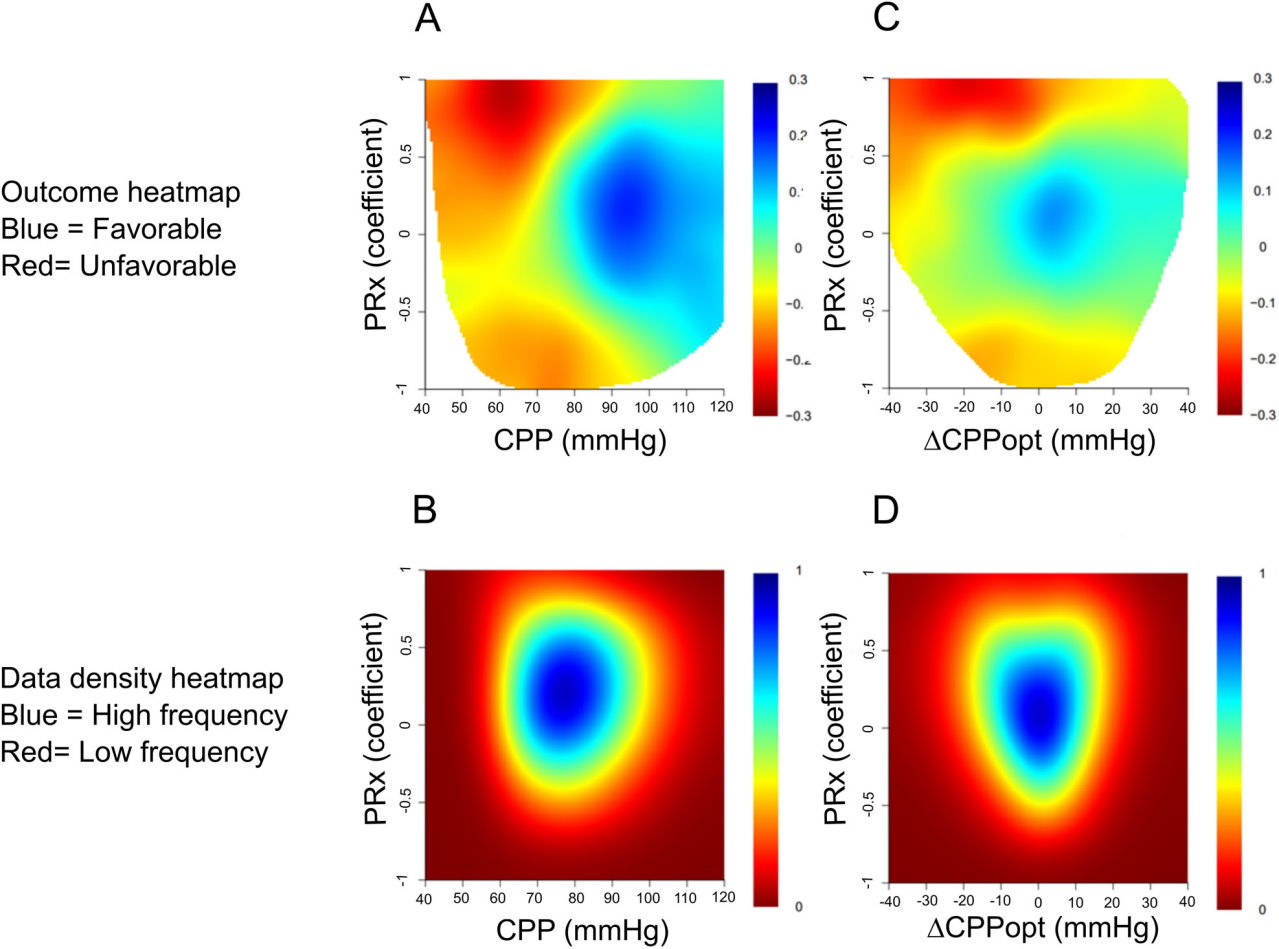
(A-D) Combined insults of PRx together with absolute CPP and ΔCPPopt the first 10 days after ictus: relation to GOS-E and data density. The upper figures demonstrate outcome heatmaps of specific PRx and CPP (A) or ΔCPPopt (C) combinations. PRx (range − 1 to + 1) was divided into 0.05 intervals, CPP (range 40-120 mm Hg) was divided into 2 mm Hg intervals, and ΔCPPopt (range −40 to +40 mm Hg) was divided into 2 mm Hg intervals. Both A and B yielded a grid of 1600 cells (40 × 40). The %GMT was calculated for all patients within each cell and was then analyzed in relation to GOS-E using the Spearman correlation. To reduce high-frequency noise, each pixel was divided into 3 × 3 smaller pixels followed by a Gaussian smoothing (standard deviation = 2). The final values for each pixel were translated into the jet color range (red to blue) with red/blue color indicating a negative/positive association with GOS-E. Pixels with less than 5 patients with at least 5 min of monitoring time were colored as white. Furthermore, a density plot was created to visualize the frequency of the %GMT for certain combinations of PRx and CPP (B) or ΔCPPopt (D). The resulting numbers were divided by the highest count within the grid to yield density values ranging from 0 to 1 for each cell in the grid, which was color-coded from red (0) to blue (1).

Abbreviations: CPP, cerebral perfusion pressure; CPPopt, optimal CPP; GMT, good monitoring time; GOS-E, Glasgow Outcome Scale-Extended; PRx, pressure reactivity index.

In the heatmap that illustrated the correlation between the %GMT of certain PRx and CPP combinations in relation to GOS-E, the combination of lower CPP with higher PRx values was more strongly associated with lower GOS-E (Figure 3A). As shown in this plot, the tolerance for lower CPP values increased with lower PRx values until a threshold of −0.50. However, for decreasing PRx below −0.50, there was a gradual reduction in the tolerance for lower CPP. These trends held true both in the early phase and the vasospasm phase (Supplemental Figure 1) but were more pronounced in the latter phase.

### Optimal Cerebral Perfusion Pressure Thresholds in Relation to Functional Outcome

In Spearman analyses of mm Hg-thresholds of ΔCPPopt, a higher %GMT of ΔCPPopt < **− **20 mm Hg in the early phase and the vasospasm phase correlated with lower GOS-E (Table 2). Similar, but slightly weaker, correlations were also found for less negative ΔCPPopt-thresholds (−15, −10, and −5 mm Hg). A higher %GMT of ΔCPPopt within mm Hg-thresholds was only significantly correlated with higher GOS-E for ± 20 mm Hg (but not 5, 10, or 15 mm Hg) in the early phase. In similar analyses of LLR/ULR-defined thresholds for ΔCPPopt, higher %GMT below the LLR (PRx > 0.60) in both the early phase and the vasospasm phase correlated with lower GOS-E (Table 3). Similar, but weaker, correlations were also found with ΔCPPopt below the LLR defined at lower PRx values (+ 0.40, + 0.20, and 0.00). A higher %GMT of ΔCPPopt within the LLR and ULR (for PRx at + 0.60 and + 0.40) also correlated with a higher GOS-E in the vasospasm phase. In similar analyses of LCIT-/UCIT-defined thresholds for ΔCPPopt, a higher %GMT below LCIT (for all PRx deteriorations; + 0.025, + 0.10, + 0.20, + 0.40, and + 0.60) in both the early phase and the vasospasm correlated with a lower GOS-E (Table 4). This was most pronounced for LCIT with PRx set at + 0.40. A higher %GMT of ΔCPPopt within the LCIT and UCIT (for all PRx thresholds except + 0.025) in the vasospasm phase correlated with a higher GOS-E.

**Table 2.** Association Between CPPopt Within/Outside mm Hg-Thresholds in the Early Phase and the Vasospasm Phase in Relation to GOS-E: A Spearman Correlation Analysis.

Variables (%GMT)	Early phase	Vasospasm phase
Below ΔCPPopt mm Hg-thresholds		
ΔCPPopt < **− **20 mm Hg	***−0***. ***14***^b^	***−0***. ***16***^b^
ΔCPPopt < **− **15 mm Hg	***−0***. ***13***^a^	***−0***. ***13***^b^
ΔCPPopt < **− **10 mm Hg	***−0***. ***13***^a^	***−0***. ***12***^a^
ΔCPPopt < **− **5 mm Hg	***−0***. ***12***^a^	***−0***. ***12***^a^
Within ΔCPPopt mm Hg-thresholds		
ΔCPPopt ± 20 mm Hg	***0***. ***12***^a^	0.06
ΔCPPopt ± 15 mm Hg	0.08	0.04
ΔCPPopt ± 10 mm Hg	0.07	0.04
ΔCPPopt ± 5 mm Hg	0.08	0.03
Above ΔCPPopt mm Hg-thresholds		
ΔCPPopt > + 20 mm Hg	0.00	0.07
ΔCPPopt > + 15 mm Hg	0.04	0.06
ΔCPPopt > + 10 mm Hg	0.06	0.07
ΔCPPopt > + 5 mm Hg	0.08	***0***. ***10***^a^

Abbreviations: CPA, cerebral pressure autoregulation; CPP, cerebral perfusion pressure; CPPopt, optimal CPP; GMT, good monitoring time; GOS-E, Glasgow Outcome Scale-Extended; PRx, pressure reactivity index.

^a^ *P* < .05, ^b^ *P* < .01, ^c^ *P* < .001. Bold and italics indicate statistical significance.

**Table 3.** Association Between CPPopt Within/Outside the LLR/ULR in the Early Phase and the Vasospasm Phase in Relation to GOS-E: A Spearman Correlation Analysis.

Variables (%GMT)	Early phase	Vasospasm phase
ΔCPPopt below LLR		
LLR (PRx) = 0.00	−0.08	***−0***. ***10***^a^
LLR (PRx) = + 0.20	−0.07	***−0***. ***12***^a^
LLR (PRx) = + 0.40	−0.08	***−0***. ***17***^c^
LLR (PRx) = + 0.60	***−0***. ***12***^a^	***−0***. ***23***^c^
CPPopt within LLR/ULR		
LLR/ULR (PRx) = 0.00	0.01	0.02
LLR/ULR (PRx) = + 0.20	0.01	0.07
LLR/ULR (PRx) = + 0.40	0.01	***0***. ***13***^b^
LLR/ULR (PRx) = + 0.60	0.05	***0***. ***20***^c^
ΔCPPopt above ULR		
ULR (PRx) = 0.00	0.10	***0***. ***10***^a^
ULR (PRx) = + 0.20	0.07	0.05
ULR (PRx) = + 0.40	0.06	−0.02
ULR (PRx) = + 0.60	0.03	−0.09

Abbreviations: CPA, cerebral pressure autoregulation; CPP, cerebral perfusion pressure; CPPopt, optimal CPP; GMT, good monitoring time; GOS-E, Glasgow Outcome Scale-Extended; LLR, lower limit of reactivity; PRx, pressure reactivity index; ULR, upper limit of reactivity.

^a^ *P* < .05, ^b^ *P* < .01, ^c^ *P* < .001. Bold and italics indicate statistical significance.

**Table 4.** Association Between CPPopt Within/Outside the LCIT/UCIT in the Early Phase and the Vasospasm Phase in Relation to GOS-E: A Spearman Correlation Analysis.

Variables (%GMT)	Early phase	Vasospasm phase
ΔCPPopt below LCIT		
LCIT (PRx) = + 0.025	***−0***. ***13***^b^	***−0***. ***15***^b^
LCIT (PRx) = + 0.10	***−0***. ***17***^b^	***−0***. ***19***^c^
LCIT (PRx) = + 0.20	***−0***. ***17***^c^	***−0***. ***21***^c^
LCIT (PRx) = + 0.40	***−0***. ***17***^c^	***−0***. ***24***^c^
LCIT (PRx) = + 0.60	***−0***. ***15***^b^	***−0***. ***23^c^***
CPPopt within LCIT/UCIT		
LCIT/UCIT (PRx) ± 0.025	0.06	0.05
LCIT/UCIT (PRx) ± 0.10	0.07	***0***. ***12***^a^
LCIT/UCIT (PRx) ± 0.20	0.08	***0***. ***14***^b^
LCIT/UCIT (PRx) ± 0.40	0.07	***0***. ***17***^c^
LCIT/UCIT (PRx) ± 0.60	0.08	***0***. ***13***^b^
ΔCPPopt above UCIT		
UCIT (PRx) = + 0.025	***0***. ***12***^a^	***0***. ***12***^a^
UCIT (PRx) = + 0.10	0.10	0.09
UCIT (PRx) = + 0.20	0.10	0.06
UCIT (PRx) = + 0.40	0.07	0.04
UCIT (PRx) = + 0.60	0.04	0.05

Abbreviations: CPA, cerebral pressure autoregulation; CPP, cerebral perfusion pressure; CPPopt, optimal CPP; GMT, good monitoring time; GOS-E, Glasgow Outcome Scale-Extended; LCIT, lower confidence interval threshold; PRx, pressure reactivity index; UCIT, upper confidence interval threshold.

^a^ *P* < .05, ^b^ *P* < .01, ^c^ *P* < .001. Bold and italics indicate statistical significance.

In the heatmap that illustrated the correlation between the %GMT of certain PRx and ΔCPPopt combinations in relation to GOS-E, the combination of negative ΔCPPopt with higher PRx values was strongly associated with lower GOS-E (Figure 3C). In particular, negative ΔCPPopt together with PRx above + 0.50 correlated with worse outcomes. In addition, there was a transition toward an unfavorable outcome when PRx went below − 0.50, particularly if ΔCPPopt was negative. These trends held true both in the early phase and the vasospasm phase (Supplemental Figure 2) but were more pronounced in the latter phase.

### CPP- and ΔCPPopt Insults in Relation to Outcome – Multiple Logistic Outcome Regressions of Unfavorable Outcome

Multiple logistic regressions of unfavorable outcome as the dependent variable were conducted with age, WFNS, DIND, and ICP in combination with different perfusion variables (%GMT of CPP < 60 mm Hg, the combination of PRx > 0.60 and CPP < 60 mm Hg, ΔCPPopt < **− **20 mm Hg, ΔCPPopt < LLR (PRx = 0.60), and ΔCPPopt LCIT (PRx=+ 0.40) as independent variables in separate regressions (Table 5). A higher %GMT of ΔCPPopt < LLR (PRx = 0.60) in the early phase and a higher %GMT of PRx > 0.60 together with CPP < 60 mm Hg in the vasospasm phase were independently associated with a higher rate of unfavorable outcome.

**Table 5.** Association Between CPP and CPPopt Targets in the Early Phase and the Vasospasm Phase in Relation to Unfavorable Outcome: A Multiple Logistic Regression.

Regression	Variables	Early phase (a)	Vasospasm phase (b)
		OR (95% CI)	OR (95% CI)
1	CPP < 60 mm Hg	1.01 (0.97-1.06)	1.06 (0.99-1.15)
2	PRx > 0.60 and CPP < 60 mm Hg	1.13 (0.96-1.41)	***1.36*** (***1.07***-***1.89***)^a^
3	ΔCPPopt < **− **20 mm Hg	1.01 (0.98-1.05)	0.98 (0.94-1.03)
4	ΔCPPopt < LLR (PRx = + 0.60)	***1.05*** (***1.01***-***1.11***)^a^	1.02 (0.99-1.06)
5	ΔCPPopt < LCIT (PRx = + 0.40)	1.02 (0.98-1.06)	1.01 (0.97-1.06)

Abbreviations: AUROC, area under receiver operating characteristics curve; CI, confidence interval; CPP, cerebral perfusion pressure; CPPopt, optimal CPP; LCIT, lower confidence interval threshold; LLR, lower limit of reactivity; OR, odds ratio; PRx, pressure reactivity index.

^a^ *P* < .05.

Age, World Federation of Neurosurgical Societies (WFNS), intracranial pressure (ICP), and delayed ischemic neurological deficit (DIND) were included as baseline variables.

Regression 1 with CPP < 60 mm Hg. 1a; AIC = 412, AUROC (95% CI) = 0.76 (0.71-0.81), Nagelkerke = 0.25. 1b; AIC = 462, AUROC (95% CI) = 0.76 (0.71-0.81), Nagelkerke = 0.25.

Regression 2 with PRx > 0.60 and CPP < 60 mm Hg. 2a; AIC = 410, AUROC (95% CI) = 0.76 (0.71-0.81), Nagelkerke = 0.25. 2b; AIC = 457, AUROC (95% CI) = 0.77 (0.72-0.81), Nagelkerke = 0.27.

Regression 3 with ΔCPPopt < 20 mm Hg. 3a; AIC = 410, AUROC (95% CI) = 0.76 (0.71-0.81), Nagelkerke = 0.25. 3b; AIC = 464, AUROC (95% CI) = 0.76 (0.71-0.80), Nagelkerke = 0.24.

Regression 4 with ΔCPPopt < LLR (PRx = + 0.60). 4a; AIC = 405, AUROC (95% CI) = 0.77 (0.72-0.82), Nagelkerke = 0.26. 4b; AIC = 463, AUROC (95% CI) = 0.76 (0.71-0.81), Nagelkerke = 0.25.

Regression 5 ΔCPPopt < LCIT (PRx = + 0.40). 5a; AIC = 409, AUROC (95% CI) = 0.76 (0.71-0.81), Nagelkerke = 0.25. 5b; AIC = 464, AUROC (95% CI) = 0.75 (0.71-0.80), Nagelkerke = 0.24. Bold and italics indicate statistical significance.

## Discussion

In this aSAH study, there was a transition toward unfavorable outcomes for decreasing CPP and ΔCPPopt. This transition depended on the concurrent cerebral pressure autoregulatory status, as the tolerance for low CPP and negative ΔCPPopt decreased for PRx above + 0.50, which likely reflects when the LLR was exceeded and ischemia occurred. Surprisingly, negative PRx below − 0.50 was unfavorable. We hypothesize that such low values reflect a state of distal vasospasm with myogenic hyperreactivity that suppressed CBF. Altogether, PRx could be used to individualize a safe CPP- and ΔCPPopt-range, but prospective validation of our findings is required. For ΔCPPopt-targets, reactivity-defined thresholds were slightly more promising as compared to the traditional mm Hg intervals.

First, we found that higher CPP was favorable in aSAH patients. Lower CPP was also more detrimental in the vasospasm phase than the early phase, that is, consistent with the idea that aSAH patients are more susceptible to developing cerebral ischemia than due to increased cerebrovascular resistance. These findings are in line with previous studies.^3,15,25^

Second, in this study, the association between CPP and outcome depended on the cerebral pressure autoregulatory status. The tolerance for low CPP decreased as PRx increased from − 0.50 to + 1.00. Furthermore, an inverse trend was seen when PRx went under − 0.50 and lower, that is, the tolerance for low CPP decreased for more negative PRx values. Thus, PRx within − 0.50 to + 0.50 appeared to be the optimal interval, while extreme values in both directions were unfavorable. This is in contrast to TBI patients, who seem to benefit also from very negative PRx.^23^ We hypothesize that the discrepant findings between aSAH and TBI cohorts could be explained by differences in cerebral pressure autoregulatory disturbances.^7^ While TBI patients often exhibit pressure passive vessels and hyperemia-induced intracranial hypertension,^26^ vasospasms both in the proximal and the distal cerebral vessels are hallmarks in the acute phase of aSAH.^2,7^ In aSAH, we suggest that extreme values of positive and negative PRx reflect vasospasm in different parts of the cerebrovascular tree. Very high PRx with relatively low CPP may indicate a state of vasospasm in the proximal cerebral vessels when there is full distal vasodilation, but the vasodilatory reserve has been exceeded at the concurrent CPP and ischemia therefore occurs. In the other scenario, very negative PRx and low CPP may indicate a state of vasospasm in the distal cerebral vessels with myogenic hyperreactivity that renders PRx negative but also suppresses CBF.^2^ Previous literature on PRx in aSAH is contradictory, as some have found PRx in isolation (without taking into account the concurrent CPP) to be associated with unfavorable outcome in aSAH,^3,27^ whereas others have found no such association.^28,29^ The heatmaps of this study illustrate that PRx may have a quadratic association with outcome, as extreme values in both directions appear detrimental, which explains why analyses of mean values might have evened out the negative effect of PRx and %GMT above threshold may not have been sensitive enough to capture the detrimental role of very negative values. In addition, the findings in this study indicated that the concurrent CPP affected the association between PRx and outcome and it is possible that differences in CPP management among studies partly explain the discrepant results in this matter in previous studies. Furthermore, also in contrast to TBI patients,^23^ the combination of elevated PRx and high CPP/ΔCPPopt was not associated with unfavorable outcomes. This could reflect that aSAH patients often are near to or even below the LLR due to vasospasm, while the ULR rarely is reached. In contrast, hyperemia in pressure passive vessels may be a more predominant pathophysiological mechanism in TBI^1,19,26^ and this could be why relatively lower CPP, between 60 and 70 mm Hg, usually is optimal on a general basis in this disease. Altogether, although CPP above 80 mm Hg and higher appears favorable in the general aSAH population level,^3,15,25^ maintaining such high targets could inflict systemic (acute respiratory distress syndrome) and cerebral (rebleeding and increased brain edema) complications.^30^ By using PRx and CPP in combined analyses, NIC management and CPP targets could be individualized for each patient for that specific moment in the acute phase.

Third, we found that negative ΔCPPopt was overall unfavorable in aSAH patients, as such insults for all definitions (mm Hg, reactivity, and confidence intervals), correlated with worse outcomes. CPPopt is an alternative perfusion target to absolute CPP thresholds that may in theory capture the autoregulatory plateau and thereby when CBF is neither ischemic nor hyperemic. Although this concept has gained acceptance and interest in TBI,^5,6,11,22^ its role in aSAH is less clear. Some aSAH studies have found a link between deviations from CPPopt with CBF^12^ and brain oxygenation,^13^ but, so far, not in relation to unfavorable outcomes.^3^ In this study, with over 400 patients, we analyzed ΔCPPopt in a novel way by assessing not only mm Hg thresholds, but also absolute PRx levels (LLR/ULR) and PRx deteriorations in relation to the nadir of the curve (LCIT/UCIT). Deviations below the LLR and the LCIT were more strongly associated with lower GOS-E and higher %GMT of being below the LLR (PRx = + 0.60) in the early phase was also the only CPPopt variable that was independently associated with unfavorable outcome. Furthermore, in the heatmap of PRx/ΔCPPopt, there was a clear transition towards an unfavorable outcome for negative ΔCPPopt when PRx exceeded approximately + 0.50, but this transition was attenuated for negative ΔCPPopt (eg, − 10 mm Hg) if PRx was not above this value. Thus, reactivity-defined thresholds appeared superior to those based on mm Hg. However, at the same time, very negative PRx values below − 0.50 were associated with unfavorable outcomes, especially for negative ΔCPPopt. The quadratic association between PRx and outcome makes the CPPopt concept less useful in aSAH, since lower PRx may not always be better. This might also explain why logistic outcome regressions yielded similar AUROCs for regressions based on ΔCPPopt targets as compared with absolute CPP targets. Overall, it remains to be determined in prospective clinical trials if CPPopt targets yield any clinical benefit over traditional CPP targets.

### Methodological Considerations

First, the study was based on a single-center cohort, which limits the external validity. Second, the tragus rather than the atrial level would have been a more accurate reference point for the measurement of ABP. Third, CPPopt could only be calculated during 54% of the GMT with CPP data, similar to previous studies.^3,31^ Fourth, we abstained from excluding physiological data with open EVD and postcraniectomy, since preliminary studies indicate that the reliability of PRx and CPPopt are preserved in these scenarios.^32–34^ Fifth, this was an exploratory study and we cannot exclude that several confounding variables to some extent explained the associations between the perfusion variables and GOS-E. Therefore, our findings need to be analyzed cautiously and prospective validation is needed. Of particular interest, it is possible that treatments such as sedation and vasopressors influenced cerebrovascular tone/reactivity, however, preliminary TBI studies suggest that these agents only exert a small effect on PRx.^35^ Sixth, we focused on the relation between cerebral perfusion variables and GOS-E in this study, however, it would be of interest to also study DIND and multimodality monitoring tools (CBF imaging, brain tissue oxygenation, and microdialysis energy metabolites) as alternative outcome measures to GOS-E in future work. Seventh, PRx and CPPopt reflect the global autoregulatory status, but these concepts may be insensitive to significant, but focal disturbances.

## Conclusions

Our study highlights that the safe CPP and ΔCPPopt range may depend on PRx, as lower CPP and negative ΔCPPopt were better tolerated within the PRx interval − 0.50 to + 0.50. Further prospective trials are needed to determine if PRx could be used to individualize NIC management. For CPPopt targets, reactivity-defined thresholds were slightly more promising as compared to the traditional mm Hg intervals. However, deviation from CPPopt thresholds yielded similar AUROCs as deviation from absolute CPP thresholds in logistic regressions of outcome and it remains to be determined in prospective clinical trials if CPPopt is superior to traditional CPP targets.

## Supplemental Material

sj-docx-1-jic-10.1177_08850666241252415 - Supplemental material for Individualized Autoregulation-Derived Cerebral Perfusion Targets in Aneurysmal Subarachnoid Hemorrhage: A New Therapeutic Avenue? Supplemental material, sj-docx-1-jic-10.1177_08850666241252415 for Individualized Autoregulation-Derived Cerebral Perfusion Targets in Aneurysmal Subarachnoid Hemorrhage: A New Therapeutic Avenue? by Teodor Mikael Svedung Wettervik, Anders Hånell, Timothy Howells, Elisabeth Ronne-Engström, Anders Lewén and Per Enblad in Journal of Intensive Care Medicine

sj-docx-2-jic-10.1177_08850666241252415 - Supplemental material for Individualized Autoregulation-Derived Cerebral Perfusion Targets in Aneurysmal Subarachnoid Hemorrhage: A New Therapeutic Avenue? Supplemental material, sj-docx-2-jic-10.1177_08850666241252415 for Individualized Autoregulation-Derived Cerebral Perfusion Targets in Aneurysmal Subarachnoid Hemorrhage: A New Therapeutic Avenue? by Teodor Mikael Svedung Wettervik, Anders Hånell, Timothy Howells, Elisabeth Ronne-Engström, Anders Lewén and Per Enblad in Journal of Intensive Care Medicine

sj-docx-3-jic-10.1177_08850666241252415 - Supplemental material for Individualized Autoregulation-Derived Cerebral Perfusion Targets in Aneurysmal Subarachnoid Hemorrhage: A New Therapeutic Avenue? Supplemental material, sj-docx-3-jic-10.1177_08850666241252415 for Individualized Autoregulation-Derived Cerebral Perfusion Targets in Aneurysmal Subarachnoid Hemorrhage: A New Therapeutic Avenue? by Teodor Mikael Svedung Wettervik, Anders Hånell, Timothy Howells, Elisabeth Ronne-Engström, Anders Lewén and Per Enblad in Journal of Intensive Care Medicine

sj-docx-4-jic-10.1177_08850666241252415 - Supplemental material for Individualized Autoregulation-Derived Cerebral Perfusion Targets in Aneurysmal Subarachnoid Hemorrhage: A New Therapeutic Avenue? Supplemental material, sj-docx-4-jic-10.1177_08850666241252415 for Individualized Autoregulation-Derived Cerebral Perfusion Targets in Aneurysmal Subarachnoid Hemorrhage: A New Therapeutic Avenue? by Teodor Mikael Svedung Wettervik, Anders Hånell, Timothy Howells, Elisabeth Ronne-Engström, Anders Lewén and Per Enblad in Journal of Intensive Care Medicine
