# Supplementary material for: Individualized Autoregulation-Derived Cerebral Perfusion Targets in Aneurysmal Subarachnoid Hemorrhage: A New Therapeutic Avenue?
Source: J Intensive Care Med. 2024 May 5;39(11):1083–92. doi: 10.1177/08850666241252415 (PMC11490071; doi:10.1177/08850666241252415)
Supplement: sj-docx-1-jic-10.1177_08850666241252415 - Supplemental material for Individualized Autoregulation-Derived Cerebral Perfusion Targets in Aneurysmal Subarachnoid Hemorrhage: A New Therapeutic Avenue? [file sj-docx-1-jic-10.1177_08850666241252415.docx]

**Supplementary table 1. Descriptive data of CPP and PRx combinations in the early phase and the vasospasm phase.**

| **Variables (%GMT)** | **Phase** | **All CPAs** | **Preserved CPA** | | | | **Lost CPA** | | | |
| --- | --- | --- | --- | --- | --- | --- | --- | --- | --- | --- |
|  |  |  | PRx < 0.00 | PRx < +0.20 | PRx < +0.40 | PRx < +0.60 | PRx > 0.00 | PRx > +0.20 | PRx > +0.40 | PRx > +0.60 |
| CPP < 60 mmHg | Early phase | 3 (1-7/0-44) | 1 (0-2/0-16) | 1 (0-3/0-22) | 2 (0-5/0-29) | 2 (1-6/0-35) | 2 (1-5/0-41) | 1 (0-4/0-40) | 1 (0-2/0-37) | 0 (0-1/0-35) |
|  | Vasospasm phase | 1 (0-3/0-100) | 0 (0-1/0-54) | 0 (0-1/0-65) | 1 (0-2/0-73) | 1 (0-2/0-82) | 1 (0-2/0-100) | 1 (0-2/0-99) | 0 (0-1/0-98) | 0 (0-1/0-97) |
| CPP 60 to 80 mmHg | Early phase | 63 (45-73/0-92) | 17 (10-28/0-56) | 31 (18-43/0-71) | 44 (28-56/0-79) | 55 (38-65/0-85) | 38 (27-51/0-86) | 24 (16-35/0-78) | 13 (8-20/0-64) | 5 (3-8/0-46) |
|  | Vasospasm phase | 40 (21-56/0-98) | 11 (5-18/0-50) | 18 (9-28/0-63) | 26 (14-37/0-76) | 33 (18-46/0-94) | 25 (11-38/0-89) | 17 (8-28/0-81) | 9 (4-18/0-64) | 4 (2-9/0-45) |
| CPP > 80 mmHg | Early phase | 26 (14-47/0-100) | 8 (4-15/0-46) | 13 (7-25/0-75) | 18 (9-33/0-98) | 22 (11-41/0-100) | 16 (9-32/0-86) | 11 (6-23/0-71) | 7 (3-13/0-49) | 3 (1-6/0-27) |
|  | Vasospasm phase | 53 (35-74/0-100) | 14 (8-22/0-56) | 23 (14-35/0-79) | 35 (21-50/0-90) | 45 (29-63/0-93) | 36 (24-51/0-91) | 26 (16-38/0-81) | 15 (9-24/0-70) | 6 (3-10/0-45) |

The numbers reflect median values (IQR/range) of the %GMT.

Preserved and lost CPA were explored according to different operational definitions as below and above 0.00, +0.20, +0.40, and +0.60, respectively, where higher values indicate a more disturbed CPA.

CPA = Cerebral pressure autoregulation. CPP = Cerebral perfusion pressure. IQR = Interquartile range. GMT = Good monitoring time. PRx = Pressure reactivity index.
