# Supplementary material for: Individualized Autoregulation-Derived Cerebral Perfusion Targets in Aneurysmal Subarachnoid Hemorrhage: A New Therapeutic Avenue?
Source: J Intensive Care Med. 2024 May 5;39(11):1083–92. doi: 10.1177/08850666241252415 (PMC11490071; doi:10.1177/08850666241252415)
Supplement: sj-docx-3-jic-10.1177_08850666241252415 - Supplemental material for Individualized Autoregulation-Derived Cerebral Perfusion Targets in Aneurysmal Subarachnoid Hemorrhage: A New Therapeutic Avenue? [file sj-docx-3-jic-10.1177_08850666241252415.docx]

**Supplementary Figure 2A-D. Combined insults of PRx together with ΔCPPopt in the early phase and the vasospasm phase: relation to GOS-E and data density**


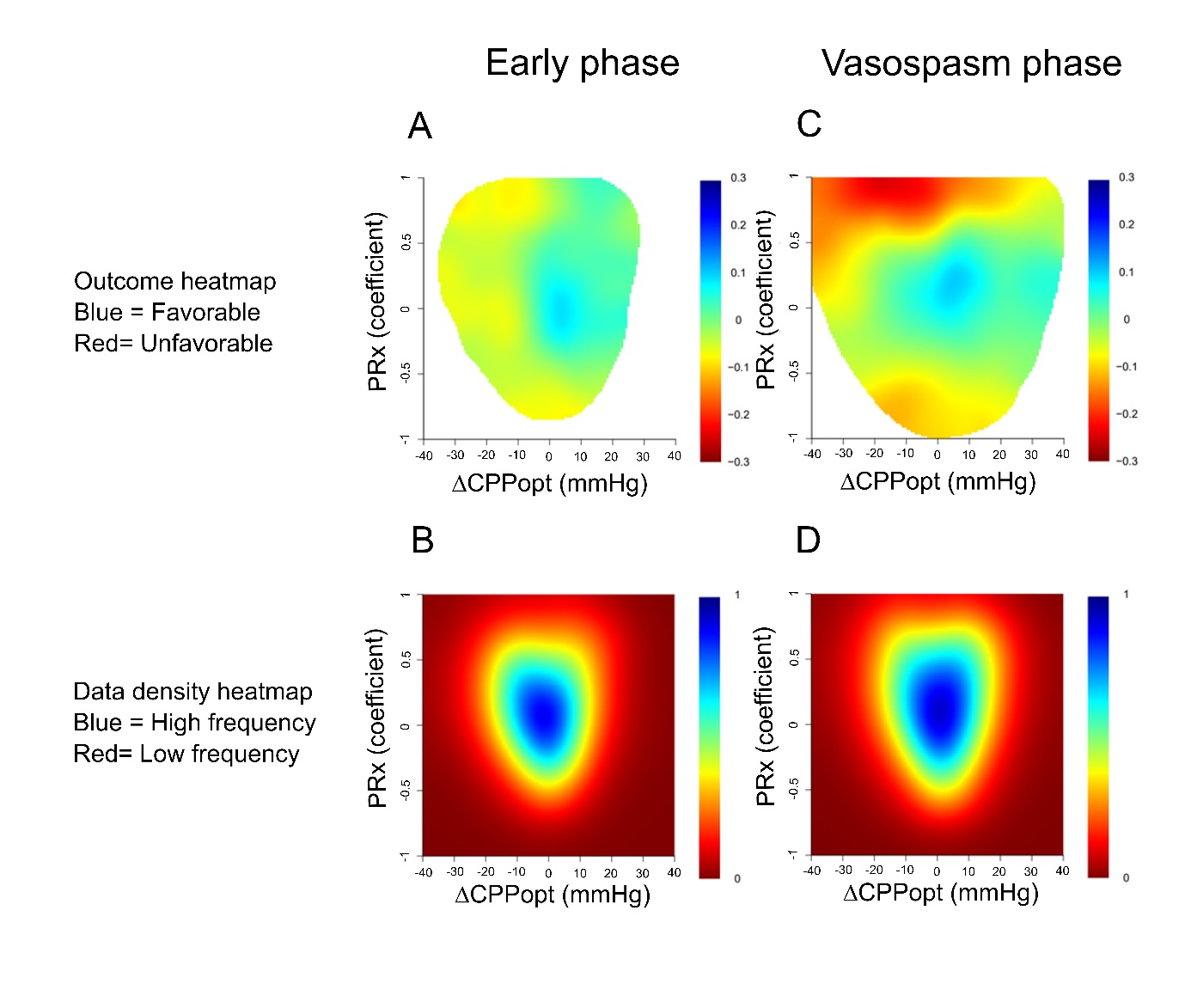


In Figure A, the %GMT for the concurrent combination of PRx and ΔCPPopt during the early phase (days 1-3) after ictus was calculated and correlated with GOS-E. The jet color range denotes the value of the correlation coefficients, where blue color indicates favorable and red color indicate unfavorable outcome. Pixels with less than five patients with five minutes of monitoring with a certain combination of PRx and ΔCPPopt were colored as white. In Figure B, the density plot illustrates the data frequency of certain PRx and ΔCPPopt combinations. The resulting numbers were divided by the highest count within the grid to yield density values ranging from 0 to 1 for each cell in the grid. The final values were then transformed to colors using the jet color scale, where blue color indicates highly frequent episodes of such PRx and ΔCPPopt combinations and red color indicates that such a PRx and ΔCPPopt combination was rare. Similar %GMT (C) and density (D) plots were conducted for the vasospasm phase (days 4-10).

CPP = Cerebral perfusion pressure. CPPopt = Optimal CPP. GMT = Good monitoring time. GOS-E = Glasgow Outcome Scale-Extended. PRx = Pressure reactivity index.
