# Supplementary material for: Individualized Autoregulation-Derived Cerebral Perfusion Targets in Aneurysmal Subarachnoid Hemorrhage: A New Therapeutic Avenue?
Source: J Intensive Care Med. 2024 May 5;39(11):1083–92. doi: 10.1177/08850666241252415 (PMC11490071; doi:10.1177/08850666241252415)
Supplement: sj-docx-4-jic-10.1177_08850666241252415 - Supplemental material for Individualized Autoregulation-Derived Cerebral Perfusion Targets in Aneurysmal Subarachnoid Hemorrhage: A New Therapeutic Avenue? [file sj-docx-4-jic-10.1177_08850666241252415.docx]

**STROBE Statement**

Checklist of items that should be included in reports of observational studies

| **Section/Topic** | Item No | Recommendation | Reported on Page No |
| --- | --- | --- | --- |
| **Title and abstract** | 1 | 1. Indicate the study’s design with a commonly used term in the title or the abstract   The abstract indicates this (page 2). |  |
|  |  | 1. Provide in the abstract an informative and balanced summary of what was done and what was found   The abstract describes this in the “Methods” and “Results” sections (page 2). |  |
| Introduction | | | |
| Background/rationale | 2 | Explain the scientific background and rationale for the investigation being reported  This is explained in the “Introduction” (page 3). |  |
| Objectives | 3 | State specific objectives, including any prespecified hypotheses  This is explained in the last paragraph of the “Introduction” (page 3). |  |
| Methods | | | |
| Study design | 4 | Present key elements of study design early in the paper  This is explained in the “Study design and patients” of the “Materials and Methods” (page 4). |  |
| Setting | 5 | Describe the setting, locations, and relevant dates, including periods of recruitment, exposure, follow-up, and data collection  This is explained in the entire “Materials and Methods” (pages 4-8). |  |
| Participants | 6 | 1. *Cohort study*—Give the eligibility criteria, and the sources and methods of selection of participants. Describe methods of follow-up   This is explained in the “Study design and patients” of the “Materials and Methods” (page 4).  *Case-control study*—Give the eligibility criteria, and the sources and methods of case ascertainment and control selection. Give the rationale for the choice of cases and controls  *Cross-sectional study*—Give the eligibility criteria, and the sources and methods of selection of participants |  |
|  |  | 1. *Cohort study*—For matched studies, give matching criteria and number of exposed and unexposed   This was not a matched study.  *Case-control study*—For matched studies, give matching criteria and the number of controls per case |  |
| Variables | 7 | Clearly define all outcomes, exposures, predictors, potential confounders, and effect modifiers. Give diagnostic criteria, if applicable  This is described in the “Statistical analysis” of the “Materials and Methods” (pages 7-8). |  |
| Data sources/measurement | 8* | For each variable of interest, give sources of data and details of methods of assessment (measurement). Describe comparability of assessment methods if there is more than one group  This is explained in the entire “Materials and Methods” (pages 4-8). |  |
| Bias | 9 | Describe any efforts to address potential sources of bias  This is described in the “Statistical analysis” of the “Materials and Methods” (pages 7-8). |  |
| Study size | 10 | Explain how the study size was arrived at  This is explained in the “Study design and patients” of the “Materials and Methods” (page 4). |  |
| Quantitative variables | 11 | Explain how quantitative variables were handled in the analyses. If applicable, describe which groupings were chosen and why  This is described in the “Statistical analysis” of the “Materials and Methods” (pages 7-8). |  |
| Statistical methods | 12 | 1. Describe all statistical methods, including those used to control for confounding   This is described in the “Statistical analysis” of the “Materials and Methods” (pages 7-8). |  |
|  |  | 1. Describe any methods used to examine subgroups and interactions   This is described in the “Statistical analysis” of the “Materials and Methods” (pages 7-8). |  |
|  |  | 1. Explain how missing data were addressed   This is described in the “Statistical analysis” of the “Materials and Methods” (pages 7-8) and the “Results” (pages 9-11). |  |
|  |  | 1. *Cohort study*—If applicable, explain how loss to follow-up was addressed   This is explained in the entire “Materials and Methods” (pages 4-8).  *Case-control study*—If applicable, explain how matching of cases and controls was addressed  *Cross-sectional study*—If applicable, describe analytical methods taking account of sampling strategy |  |
|  |  | 1. Describe any sensitivity analyses   None. |  |

| **Section/Topic** | Item No | Recommendation | Reported on Page No |
| --- | --- | --- | --- |
| Results | | | |
| Participants | 13* | 1. Report numbers of individuals at each stage of study—eg numbers potentially eligible, examined for eligibility, confirmed eligible, included in the study, completing follow-up, and analysed   This is explained in the “Study design and patients” of the “Materials and Methods” (page 4). |  |
|  |  | 1. Give reasons for non-participation at each stage   This is explained in the “Study design and patients” of the “Materials and Methods” (page 4). |  |
|  |  | 1. Consider use of a flow diagram   No flow diagram. |  |
| Descriptive data | 14* | 1. Give characteristics of study participants (eg demographic, clinical, social) and information on exposures and potential confounders   This is described in the “Results” (pages 9-11) |  |
|  |  | 1. Indicate number of participants with missing data for each variable of interest   This is described in the “Statistical analysis” of the “Materials and Methods” (pages 7-8) and the “Results” (pages 9-11). |  |
|  |  | 1. *Cohort study*—Summarise follow-up time (eg, average and total amount)   This is described in the entire “Materials and Methods” (pages 4-8). |  |
| Outcome data | 15* | *Cohort study*—Report numbers of outcome events or summary measures over time  This is described in the “Materials and Methods” (pages 4-8). |  |
|  |  | *Case-control study—*Report numbers in each exposure category, or summary measures of exposure |  |
|  |  | *Cross-sectional study—*Report numbers of outcome events or summary measures |  |
| Main results | 16 | 1. Give unadjusted estimates and, if applicable, confounder-adjusted estimates and their precision (eg, 95% confidence interval). Make clear which confounders were adjusted for and why they were included   This is explained in the “Results” (pages 9-11). |  |
|  |  | 1. Report category boundaries when continuous variables were categorized   Outcome was dichotomized into favorable and unfavorable outcome (GOS-E 5-8/1-4). |  |
|  |  | 1. If relevant, consider translating estimates of relative risk into absolute risk for a meaningful time period   None. |  |
| Other analyses | 17 | Report other analyses done—eg analyses of subgroups and interactions, and sensitivity analyses  None. |  |
| Discussion | | | |
| Key results | 18 | Summarise key results with reference to study objectives  This is described in the first paragraph of the “Discussion” (page 12). |  |
| Limitations | 19 | Discuss limitations of the study, taking into account sources of potential bias or imprecision. Discuss both direction and magnitude of any potential bias  This is considered in the entire “Discussion” (pages 12-14). |  |
| Interpretation | 20 | Give a cautious overall interpretation of results considering objectives, limitations, multiplicity of analyses, results from similar studies, and other relevant evidence  This is considered in the entire “Discussion” (pages 12-14). |  |
| Generalisability | 21 | Discuss the generalisability (external validity) of the study results  This is explained in the “Conclusions” (page 15). |  |
| Other Information | | | |
| Funding | 22 | Give the source of funding and the role of the funders for the present study and, if applicable, for the original study on which the present article is based  No conflict of interest. |  |

**Give information separately for cases and controls in case-control studies and, if applicable, for exposed and unexposed groups in cohort and cross-sectional studies.*

**Note:** An Explanation and Elaboration article discusses each checklist item and gives methodological background and published examples of transparent reporting. The STROBE checklist is best used in conjunction with this article (freely available on the Web sites of PLoS Medicine at http://www.plosmedicine.org/, Annals of Internal Medicine at http://www.annals.org/, and Epidemiology at http://www.epidem.com/). Information on the STROBE Initiative is available at www.strobe-statement.org.
